# Epidemiology of human papillomavirus-related oropharyngeal cancer in a classically low-burden region of southern Europe

**DOI:** 10.1038/s41598-020-70118-7

**Published:** 2020-08-06

**Authors:** M. Mena, J. Frias-Gomez, M. Taberna, B. Quirós, S. Marquez, O. Clavero, A. Baena, B. Lloveras, M. Alejo, X. León, J. García, R. Mesía, O. Bermejo, T. Bonfill, A. Aguila, M. Guix, R. Hijano, M. A. Pavón, M. Torres, S. Tous, R. Clèries, L. Alemany

**Affiliations:** 1 Cancer Epidemiology Research Program, Institut Català d’Oncologia (ICO), L’Hospitalet de Llobregat, Av. Gran Via de L’Hospitalet 199-203, 08908 Barcelona, Spain; 2 Epidemiology, Public Health, Cancer Prevention and Palliative Care Program, Institut d’Investigació Biomèdica de Bellvitge (IDIBELL), L’Hospitalet de Llobregat, Barcelona, Spain; 3 University of Barcelona, Barcelona, Spain; 4 Department of Medical Oncology, ICO, L’Hospitalet de Llobregat, Barcelona, Spain; 5 Program of Molecular Mechanisms and Experimental Therapy in Oncology (ONCOBELL), IDIBELL, L’Hospitalet de Llobregat, Barcelona, Spain; 6 Tobacco Control Unit, WHO Collaborating Centre for Tobacco Control, ICO, IDIBELL, L’Hospitalet de Llobregat, Barcelona, Spain; 7 Department of E-Health, Faculty of Health Sciences, Universitat Oberta de Catalunya, Barcelona, Spain; 8 Department of Pathology, Hospital del Mar, Barcelona, Spain; 9 Department of Pathology, Hospital General de L’Hospitalet, Barcelona, Spain; 10 Department of Otorhinolaryngology, Hospital de Sant Pau, Barcelona, Spain; 11 Centro de Investigación Biomédica en Red de Bioingeniería, Biomateriales y Nanomedicina (CIBER-BBN), Instituto de Salud Carlos III, Madrid, Spain; 12 Department of Medical Oncology, ICO, B-ARGO Group, Badalona, Barcelona Spain; 13 Department of Plastic Surgery, Bellvitge University Hospital, L’Hospitalet de Llobregat, Barcelona, Spain; 14 Department of Medical Oncology, Corporació Sanitària Parc Taulí, Sabadell, Barcelona Spain; 15 Department of Otorhinolaryngology, Corporació Sanitària Parc Taulí, Sabadell, Barcelona Spain; 16 Department of Medical Oncology, Hospital del Mar, Barcelona, Spain; 17 Department of Otorhinolaryngology, Hospital del Mar, Barcelona, Spain; 18 Pla Director d’Oncologia, ICO, IDIBELL, L’Hospitalet de Llobregat, Barcelona, Spain; 19 Dept. Ciències Clíniques, Universitat de Barcelona, Barcelona, Spain; 20 Centro de Investigación Biomédica en Red: Epidemiología y Salud Pública (CIBERESP), Instituto de Salud Carlos III, Madrid, Spain

**Keywords:** Cancer epidemiology, Head and neck cancer, Tumour virus infections

## Abstract

The incidence of human papillomavirus (HPV)-related oropharyngeal cancer is increasing in some regions. Nevertheless, the epidemiology of this disease has not been extensively investigated in southern Europe. We conducted a retrospective cohort study of patients diagnosed with primary oropharyngeal cancer from 1991 to 2016. Cancer tissues underwent histopathological evaluation, DNA quality control, HPV-DNA detection and p16^INK4a^ immunohistochemistry. Data were collected from medical records. Factors associated with HPV positivity and time trends were evaluated with multivariable Bayesian models. The adjusted prevalence of HPV-related cases in 864 patients with a valid HPV-DNA result was 9.7%, with HPV-DNA/p16^INK4a^ double positivity being considered. HPV-related oropharyngeal cancer was likely to occur in non-smokers and non-drinkers, to be located in the tonsil or diagnosed at advanced stages. Time-trend analysis showed an increasing risk of HPV-related oropharyngeal cancer in the most recent periods (5-year period increase of 30%). This increase was highest and with a clear increasing trend only in the most recent years (2012–2016). The prevalence of HPV-related oropharyngeal cancer started to sharply increase in the most recent years in our setting, as occurred two decades ago in areas where most oropharyngeal cancer cases are currently HPV-related. Our results provide a comprehensive assessment of the epidemiological landscape of HPV-related oropharyngeal cancer in a region of southern Europe.

## Introduction

The epidemiology of oropharyngeal cancer (OPC) has dramatically changed in the last two decades after the incursion of human papillomavirus (HPV) in the etiological arena of the disease^1^. Currently, 29,000 new HPV-related OPC cases (representing 30% of the total OPC) are estimated to occur every year worldwide^2^, with marked differences between geographical regions. HPV-related OPC is increasing in some regions of the world such as North America^3^ and northern Europe^4–7^, where most OPC cases are currently HPV-related. The increasing trends are thought to be related to a decrease in smoking and increased numbers of sexual partners and oral sex practices^8, 9^. The role of HPV in OPC has important clinical implications given the distinct nature and marked prognostic differences between HPV-related and non-related patients^8, 9^. Nevertheless, the epidemiology of the disease in southern Europe, where current estimates of HPV prevalence are low (9–24%)^2,10^, has not been extensively investigated. In Spain, only two studies on HPV involvement in selected OPC cases were available^11,12^ until our group recently reported the results of a multicenter retrospective cohort of 788 patients with primary OPC consecutively diagnosed between 1991 and 2013^13^. The study identified double positivity for HPV-DNA/p16^INK4a^ as the biomarker with strongest diagnostic accuracy and prognostic value for HPV-related OPC patients and found an HPV prevalence of 7.4% when considering HPV-DNA/p16^INK4a^ double positivity^13^. A recent Italian study also evaluated the role of HPV in patients with newly diagnosed OPC during the period 2000–2018, reporting a prevalence of HPV-driven OPC of 32.3% and a higher prevalence in the most recent years^14^.

In this study, we aimed to provide an updated assessment of the time trends of HPV prevalence estimates in OPC, as well as to evaluate further its epidemiological features compared with other high-burden regions.

## Methods

### Study design and population

We designed a retrospective cohort study of all new patients diagnosed with primary OPC in four hospitals in Catalonia (Spain) from 1991 to 2013 (Catalan Institute of Oncology-Bellvitge Hospital, Hospital del Mar, Hospital Parc Taulí and Hospital de la Santa Creu i Sant Pau). We also included all patients diagnosed with primary OPC in the latter setting through 2016.

The procedures followed have been previously described^10^. Briefly, cases were identified from medical records/pathology reports of the centers of origin. We included cases fulfilling the following criteria: diagnosis of primary invasive cancer of the oropharynx, any histology, codes from the International Classification of Diseases for Oncology version 3: Tonsil-C09, C02.4, Base of tongue-C01 and Others (soft palate-C05.1, uvula-C05.2, vallecula, glossoepiglottic fold, lateral and posterior wall of the oropharynx, overlapping lesion of the oropharynx and oropharynx unspecified-C10, Waldeyer ring-C14.2); and available access to medical records on demographic, toxic habits and clinical and follow-up information. Formalin-fixed paraffin embedded (FFPE) primary tumour samples from the diagnosis prior to treatment were retrieved when available.

### FFPE block processing and histopathological evaluation

All specimens processing was centralized at the Catalan Institute of Oncology (ICO). FFPE blocks were re-embedded whenever necessary. The first and last sections were used for histopathological evaluation after hematoxylin and eosin (H&E) staining. Two in-between sections were used for HPV-DNA testing and genotyping; one additional slide was obtained to assess p16^INK4a^ expression, which was performed at Hospital General de l’Hospitalet (Spain) under the manufacturer’s standards (Roche mtm Laboratories AG IHC. Heidelberg, Germany). A strong and diffuse nuclear and cytoplasmic staining of > 70% of the cancer tissue was considered p16^INK4a^-positive^15^. Since our previous study showed high diagnostic accuracy and prognostic value for double positivity for HPV-DNA/p16^INK4a^^13^, which is an easier diagnostic algorithm to implement in clinical practice than HPV-mRNA testing, we did not further use HPV-mRNA positivity for the analyses herein presented. A block was classified as adequate for HPV testing if invasive cancer was observed in the two H&E stained sections of the specimen.

Pathology review was based on the WHO pathological criteria for head and neck cancer and was performed blind with respect to the original local diagnosis and HPV-DNA/p16^INK4a^ results, following a pre-established algorithm for diagnostic consensus involving three pathologists, as reported elsewhere^10^.

### HPV-DNA detection and genotyping

We used a PCR with the consensus primers SPF10 PCR and a DNA enzyme immunoassay (DEIA) to test for the presence of HPV-DNA^10,13^. HPV genotyping was performed using a reverse hybridization line probe assay (LiPA25_v1) on all samples testing positive for viral DNA, targeting 25 HPV types with different oncogenic risk (Laboratory Biomedical Products Rijswijk, The Netherlands). DNA quality was evaluated in all HPV-DNA negative samples by testing for the *tubulin*-β gene. All DEIA and LiPA25_v1 assays were performed at the ICO.

### Statistical analyses

OPC samples testing negative for both viral and human DNA were excluded from the analyses. Descriptive, bivariate and unconditional logistic regression analyses were performed to identify independent factors associated with HPV etiological involvement in OPC according to double positivity for HPV-DNA/p16^INK4a^ and p16^INK4a^-positivity only. Crude and adjusted odds ratios and their 95% credibility intervals (CI, the Bayesian version of the frequentist confidence intervals but calculated using the posterior distribution of the model’s parameters), were estimated. Moreover, crude and adjusted prevalences were estimated, the latter to account for other covariates that may have an effect on the prevalence estimates. Adjusted prevalences were calculated with R “prediction” function of “prediction” package^16^ based on the adjusted models. Histological variables were not included in the multivariable analyses as they were considered to be intermediate variables in the carcinogenic process, as previously described^17^. Bayesian methodology was chosen in all analyses with non-informative prior distribution due to lack of data on the distribution of the parameters^18,19^ and, moreover, because Bayesian methodology is useful to (1) avoid model fitting problems in parameter estimation due to small counts, and (2) produce robust estimators^20^.

To more specifically assess time trends in HPV-related OPCs in Spain, a log-binomial regression model was defined for double positivity for HPV-DNA/p16^INK4a^ and prevalence risk ratios (RR) were obtained, with their 95% CI. Patients from 1991 were included in the first 5-year period (1992–1996) in order not to lose sample size. Multivariable models were adjusted for confounders: age, 5-year periods, gender, tobacco/alcohol consumption, subsite and tumour stage.

All analyses were performed using the “bayesglm” function of the “arm” package^19^ in R. All possible interactions between variables were assessed and stratified by anatomical subsite.

### Ethics approval and consent to participate

This study was performed in accordance with the Declaration of Helsinki. The study had formal approval by the ethics committees of the four participating hospitals (i.e. Catalan Institute of Oncology-ICO-Hospital Universitari de Bellvitge, Hospital de la Santa Creu i Sant Pau, Hospital del Mar and Hospital Parc Taulí). Adequate measures to ensure data protection, confidentiality, patients’ privacy and anonymization were taken into account in compliance with European and Spanish current laws and regulations. Informed consent was not available due to the retrospective design of the study and the large proportion of deceased and untraceable patients. Thus, and in accordance to current regulations, informed consent was waved off by the ethics committees for patients diagnosed up to 2013. Informed consent was obtained for patients diagnosed in 2014–2017.

## Results

Supplementary Fig. 1 describes the workflow of the OPC targeted cases, samples collected, processed, tested and finally included in the statistical analyses. A total of 76 OPC patients diagnosed in Hospital de la Santa Creu i Sant Pau between 2013 and 2016 were added to the initial retrospective cohort of 788 patients^13^.

### HPV prevalence and factors associated with HPV positivity in OPC

Table 1 shows the demographic and clinical characteristics of the 864 patients included in the analysis, as well as the crude and adjusted prevalences and odds ratios for double positivity for HPV-DNA/p16^INK4a^. Patients had a mean age at diagnosis of 60 years; they were mostly male (88.9%), heavy smokers (74.4%) and heavy drinkers (49.7%). The most common anatomical subsite was the tonsil (40.5%). Most tumours (63.3%) were squamous cell carcinoma (SCC) with conventional keratinizing morphological features and the most common stage was IVa according to the 7th TNM edition (45.6%). The adjusted prevalence of HPV-related patients was 9.7%.

**Table 1:** Association of demographic and clinical characteristics of OPC patients and HPV double positivity for HPV-DNA/p16^INK4a^.

Characteristics	OPC samples (n = 864) No. (%)^a^	HPV-DNA detection AND p16^INK4a^ high expression (n = 78)			
		Crude prevalence No. (%)^b^	Adjusted prevalence^c^ (%)	Crude OR [95% CI]	Adjusted OR^c^ [95% CI]
*Age at diagnosis (years)*					
≤ 60	456 (52.9)	46 (10.1)	13.1	1.4 [0.8–2.2]	**3.0 [1.6–5.7]**
> 60	406 (47.1)	31 (7.6)	6.8	Ref	Ref
Mean age at diagnosis (SD)	60.3 (10.7)	58.7 (13.4)			
Age range	28–93	28–93			
*Gender*					
Male	766 (88.9)	57 (7.4)	9.3	Ref	Ref
Female	96 (11.1)	21 (21.9)	11.1	**3.4 [2.0–5.9]**	1.3 [0.6–2.8]
*Center*^*d*^					
H Mar	100 (11.6)	6 (6.0)		Ref	
H ICO-Bellvitge	241 (27.9)	18 (7.5)		1.2 [0.5–2.9]	
H Parc Taulí	84 (9.7)	5 (6.0)		1.0 [0.3–2.9]	
H Sant Pau	439 (50.8)	49 (11.2)		1.9 [0.8–4.2]	
*Period of diagnosis*					
1991–1996	140 (16.2)	7 (5.0)	7.7	Ref	Ref
1997–2001	97 (11.2)	2 (2.1)	2.7	0.4 [0.1–1.7]	0.2 [0.1–1.1]
2002–2006	221 (25.6)	16 (7.2)	8.6	1.4 [0.6–3.2]	1.2 [0.4–3.1]
2007–2011	257 (29.7)	21 (8.2)	8.4	1.6 [0.7–3.6]	1.1 [0.4–2.9]
2012–2016	149 (17.2)	32 (21.5)	18.3	**4.9 [2.2–10.7]**	**4.3 [1.6–11.4]**
*Tobacco use*					
Non-smoker	90 (11.2)	36 (40.0)	23.4	**15.8 [8.2–28.3]**	**9.4 [4.3–20.7]**
< 20 cigarettes/day	116 (14.4)	19 (16.4)	11.1	**4.6 [2.5–8.7]**	**2.7 [1.3–5.7]**
≥ 20 cigarettes/day	598 (74.4)	23 (3.8)	5.3	Ref	Ref
*Alcohol consumption*					
Non-drinker	161 (20.0)	46 (28.6)	14.7	**27.0[11.4–64.2]**	**10.2 [3.8–27.5]**
< 100 g/day	245 (30.4)	27 (11.0)	12.4	**8.3 [3.4–20.2]**	**7.8 [3.0–20.1]**
≥ 100 g/day	401 (49.7)	5 (1.2)	2.6	Ref	Ref
*Subsite*					
Tonsil	350 (40.5)	53 (15.1)	14.1	**5.6 [2.8–11.1]**	**5.2 [2.4–11.5]**
BOT	189 (21.9)	16 (8.5)	7.5	**2.8 [1.3–6.3]**	1.9 [0.8–4.9]
Tonsil & BOT	19 (2.2)	0 (0.0)	2.2	0.4 [0.0–7.6]	0.4 [0.0–8.8]
Others^e^	306 (35.4)	9 (2.9)	4.7	Ref	Ref
*Stage (7th edition TNM)*					
I&II	183 (21.3)	6 (3.3)	4.4	Ref	Ref
III	184 (21.4)	19 (10.3)	12.2	**3.1 [1.3–7.3]**	**4.7 [1.7–12.6]**
IVa	393 (45.6)	49 (12.5)	11.6	**3.8 [1.7–8.5]**	**4.3 [1.7–10.6]**
IVb	82 (9.5)	4 (4.9)	6.1	1.4 [0.4–4.5]	1.6 [0.4–6.0]
IVc	19 (2.2)	0 (0.0)	2.5	0.3 [0.0–6.8]	0.5 [0.0–13.6]
*Histology*^d^					
SCC Conventional keratinizing	547 (63.3)	20 (3.7)		Ref	
SCC Conventional non-keratinizing	234 (27.1)	28 (12.0)		**3.4 [1.9–6.0]**	
SCC Basaloid, papillary, exophitic	73 (8.4)	29 (39.7)		**16.2 [8.6–30.5]**	
SCC Sarcomatoid	3 (0.3)	0 (0.0)		0.7 [0.0–28.3]	
Non-SCC^f^	7 (0.8)	1 (14.3)		3.0 [0.4–24.3]	
Total	864	78 (9.0)	9.7		

*OPC* Oropharyngeal carcinoma, *SD* Standard deviation, *H* Hospital, *SCC* Squamous cell carcinoma, *BOT* base of tongue, *CI* credibility interval.

^a^Column percentage. ^b^Row percentage. ^c^Adjusted by age at diagnosis, gender, period of diagnosis, subsite, tobacco and alcohol consumption and stage. ^d^Not considered in the multivariable model. ^e^Others include: Soft palate-C05.1, Uvula-C05.2, Vallecula, Glossoepiglottic fold, lateral and posterior wall of the oropharynx, overlapping lesion of the oropharynx and oropharynx unspecified-C10, Waldeyer ring-C14.2. ^f^Non SCC include: 4 undifferentiated (1 of them HPV double positive) and three neuroendocrine carcinomas. In bold those estimates showing a clear association with HPV-DNA/p16^INK4a^ positivity (i.e. credibility intervals do not contain 1.0).

After adjustment for confounders, HPV-related patients were more likely to be younger than 60 years, non-smokers and non-drinkers, to have tonsillar carcinoma, and to be diagnosed in most recent periods or with stages III–IVa (Table 1). The equivalent results for p16^INK4a^ positivity alone are presented in supplementary Table 1.

### HPV prevalence and factors associated with HPV positivity by anatomical subsite

The associations of HPV-positivity with the demographic and clinical characteristics of OPC patients were also examined, stratified by three major anatomical subsites (tonsil, base of tongue and others, Table 2). None of the associations observed in the multivariable analysis for OPC including all sites were observed for subsites other than the tonsil and base of tongue. Crude and adjusted HPV prevalences stratified by the three major anatomical sites are presented for HPV-DNA/p16^INK4a^ double-positivity and p16^INK4a^-positivity alone in supplementary Tables 3 and 4, respectively.

**Table 2:** Association of demographic and clinical characteristics of OPC patients and HPV double positivity for HPV-DNA/p16^INK4a^ stratified by the three major anatomical sites (tonsil / base of tongue/others).

Characteristics	HPV-DNA detection AND high p16^INK4a^ expression								
	Tonsil samples (n = 350) Prevalence n/N (%)	Crude OR [95% CI]	Adjusted OR^a^ [95% CI]	BOT samples (n = 189) Prevalence n/N (%)	Crude OR [95% CI]	Adjusted OR^a^ [95% CI]	Others Samples^b^ (n = 306) Prevalence n/N (%)	Crude OR [95% CI]	Adjusted OR^a^ [95% CI]
*Age at diagnosis (years)*									
≤ 60	30/182 (16.5)	1.2 [0.7–2.2]	**3.5 [1.5–7.8]**	12/100 (12.0)	**3.4 [1.1–11.2]**	3.9 [0.9–17.4]	4/163 (2.5)	0.7 [0.2–2.5]	0.9 [0.2–3.6]
> 60	23/168 (13.7)	Ref	Ref	3/88 (3.4)	Ref	Ref	5/142 (3.5)	Ref	Ref
*Gender*									
Male	40/302 (13.2)	Ref	Ref	10/168 (6.0)	Ref	Ref	7/279 (2.5)	Ref	Ref
Female	13/47 (27.7)	**2.4 [1.2–4.9]**	1.2 [0.5–3.0]	6/21 (28.6)	**5.6 [1.8–16.9]**	1.7 [0.3–8.2]	2/26 (7.7)	2.7 [0.6–12.8]	1.1 [0.2–6.1]
*Center*^*c*^									
H Mar	5/49 (10.2)	Ref		1/22 (4.5)	Ref		0/27 (0.0)	Ref	
H ICO-Bellvitge	15/108 (13.9)	1.3 [0.5–3.5]		3/70 (4.3)	0.9 [0.2–5.5]		0/51 (0.0)	0.5 [0.0–20.1]	
H Parc Taulí	5/39 (12.8)	1.2 [0.4–3.9]		0/13 (0.0)	0.3 [0.0–7.9]		0/27 (0.0)	0.7 [0.0–29.9]	
H Sant Pau	28/154 (18.2)	1.8 [0.7–4.5]		12/84 (14.3)	3.3 [0.6–16.7]		9/201 (4.5)	9.4 [0.4–204.9]	
*Period of diagnosis*									
1991–1996	4/32 (12.5)	Ref	Ref	1/31 (3.2)	Ref	Ref	2/76 (2.6)	Ref	Ref
1997–2001	2/46 (4.3)	0.4 [0.1–1.6]	0.3 [0.0–1.4]	0/18 (0.0)	0.3 [0.0–6.4]	0.3 [0.1–8.3]	0/31 (0.0)	0.3 [0.0–6.0]	0.3 [0.0–6.7]
2002–2006	10/97 (10.3)	0.8 [0.3–2.41]	0.9 [0.2–3.4]	4/59 (6.8)	1.5 [0.3–7.7]	1.8 [0.3–10.7]	2/57 (3.5)	1.3 [0.2–6.9]	1.6 [0.3–9.6]
2007–2011	15/109 (13.8)	1.1 [0.4–3.2]	0.8 [0.2–2.8]	3/49 (6.1)	1.4 [0.3–7.3]	2.9 [0.4–19.0]	3/92 (3.3)	1.2 [0.3–5.6]	1.0 [0.2–5.0]
2012–2016	22/66 (33.3)	**3.4 [1.2–9.6]**	**4.1 [1.1–15.2]**	8/32 (25.0)	**6.9 [1.5–31.9]**	**10.3 [1.6–67.0]**	2/50 (4.0)	1.5 [0.3–7.9]	1.4 [0.2–8.2]
*Tobacco use*									
Non-smoker	24/46 (52.2)	**14.7 [6.9–31.4]**	**13.3 [4.8–36.9]**	8/21 (38.1)	**15.5 [4.5–52.7]**	4.6 [0.9–22.2]	4/22 (18.2)	**9.2 [2.1–37.0]**	5.7 [0.9–35.8]
< 20 cigarettes/day	14/54 (25.9)	**4.7 [2.2–10.3]**	**2.6 [1.1–6.6]**	4/18 (22.2)	**6.9 [1.7–27.5]**	3.7 [0.7–20.1]	1/40 (2.5)	1.2 [0.2–7.8]	1.2 [0.2–8.5]
≥ 20cigarettes/day	15/235 (6.4)	Ref	Ref	4/135 (3.0)	Ref	Ref	4/214 (1.9)	Ref	Ref
*Alcohol consumption*									
Non-drinker	31/91 (34.1)	**19.7 [6.8–57.2]**	**10.4 [3.1–35.1]**	11/31 (35.5)	**26.4 [5.6–124.5]**	**10.0 [1.6–63.3]**	4/37 (10.8)	**9.6 [1.8–51.2]**	4.6 [0.6–36.7]
< 100 g/day	19/95 (20.0)	**9.5 [3.2–28.2]**	**10.4 [3.2–34.4]**	4/58 (6.9)	3.5 [0.7–18.1]	2.8 [0.5–16.3]	4/85 (4.7)	4.0 [0.8–20.6]	3.7 [0.7–19.9]
≥ 100 g/day	3/151 (2.0)	Ref	Ref	1/87 (1.1)	Ref	Ref	1/154 (0.6)	Ref	Ref
*Stage (7th edition TNM)*									
I&II	6/89 (6.7)	**Ref**	Ref	0/17 (0.0)	0.2 [0.0–3.5]	0.5 [0.0–12.5]	0/74 (0.0)	0.2 [0.0–3.0]	0.2 [0.0–3.1]
III	13/72 (18.1)	**2.7 [1.1–7.0]**	**5.0 [1.6–15.5]**	4/38 (10.5)	Ref	Ref	2/72 (2.8)	Ref	Ref
IVa	31/152 (20.4)	**3.2 [1.4–7.4]**	**3.4 [1.3–9.2]**	12/100 (12.0)	1.4 [0.4–4.6]	1.5 [0.3–7.2]	6/130 (4.6)	1.8 [0.4–7.4]	1.8 [0.4–8.1]
IVb	3/27 (11.1)	1.5 [0.4–5.8]	1.2 [0.3–5.5]	0/28 (0.0)	0.1 [0.0–2.3]	0.2 [0.0–5.4]	1/24 (4.2)	1.5 [0.2–11.6]	2.1 [0.2–19.2]
IVc	0/7 (0.0)	0.4 [0.0–8.5]	0.6 [0.0–23.1]	0/6 (0.0)	0.4 [0.0–8.5]	0.3 [0.0–9.1]	0/6 (0.0)	0.6 [0.0–22.9]	0.7 [0.0–39.9]
*Histology*^*c*^									
SCC Conventional keratinizing	15/221 (6.8)	**Ref**		3/114 (2.6)	Ref		2/199 (1.0)	Ref	
SCC Conventional non-keratinizing	18/88 (20.5)	**3.3 [1.6–6.8]**		5/55 (9.1)	2.6 [0.7–9.3]		5/87 (5.7)	**4.1 [1.1–16.7]**	
SCC Basaloid, papillary, exophitic	20/36 (55.6)	**15.8 [7.0–36.0]**		7/16 (43.8)	**19.5 [5.1–74.2]**		2/19 (10.5)	**6.9 [1.1–41.9]**	
SCC Sarcomatoid	0/1 (0.0)	0.8 [0.0–40.5]		0/1 (0.0)	0.8 [0.0–61.3]		0/1 (0.0)	0.9 [0.0–90.6]	
Non-SCC^d^	0/4 (0.0)	0.5 [0.0–13.9]		1/3 (33.3)	7.8 [0.7–93.2]		-	-	
Total	53/350 (15.1)			16/189 (8.5)			9/306 (2.9)		

*OPC* oropharyngeal carcinoma, *H* hospital, *SCC* squamous cell carcinoma, *BOT* base of tongue, *CI* credibility interval.

^a^Adjusted by age, gender, period of diagnosis, subsite, tobacco and alcohol consumption and stage. ^b^Others include: Soft palate-C05.1, Uvula-C05.2, Vallecula, Glossoepiglottic fold, laterall and posterior wall of the oropharynx, overlapping lesion of the oropharynx and oropharynx unspecified-C10, Waldeyer ring-C14.2. ^c^Not considered in the multivariable model. ^d^Non SCC include: 4 undifferentiated (1 of them HPV double positive) and three neuroendocrine carcinomas. In bold those estimates showing a clear association with HPV-DNA/p16^INK4a^ positivity (i.e. credibility intervals do not contain 1.0).

**Table 3:** HPV type-specific prevalence by anatomical site according to HPV-DNA and HPV-DNA/p16^INK4a^ positivity.

Genotype	HPV-DNA	HPV-DNA/ p16^INK4a^
OPC all sites	Prevalence N (%)	Prevalence N (%)
HPV16	83 (82.2)	69 (88.5)
HPV18	2 (2.0)	1 (1.3)
HPV31	1 (1.0)	0 (0.0)
HPV33	6 (5.9)	4 (5.1)
HPV35	4 (4.0)	4 (5.1)
HPV51	1 (1.0)	0 (0.0)
HPV58	1 (1.0)	0 (0.0)
HPVX	3 (3.0)	0 (0.0)
Total	101 (100.0)	78 (100.0)
*Tonsil*		
HPV16	49 (79.0)	45 (84.9)
HPV18	2 (3.2)	1 (1.9)
HPV31	0 (0.0)	0 (0.0)
HPV33	6 (9.7)	4 (7.6)
HPV35	3 (4.8)	3 (5.7)
HPV51	0 (0.0)	0 (0.0)
HPV58	0 (0.0)	0 (0.0)
HPVx	2 (3.2)	0 (0.0)
Total	62 (100.0)	53 (100.0)
*Base of tongue*		
HPV16	18 (85.7)	15 (93.7)
HPV18	0 (0.0)	0 (0.0)
HPV31	1 (4.8)	0 (0.0)
HPV33	0 (0.0)	0 (0.0)
HPV35	1 (4.8)	1 (6.3)
HPV51	0 (0.0)	0 (0.0)
HPV58	1 (4.8)	0 (0.0)
HPVx	0 (0.0)	0 (0.0)
Total	21 (100.0)	16 (100.0)
*Others*		
HPV16	16 (88.9)	9 (100.0)
HPV18	0 (0.0)	0 (0.0)
HPV31	0 (0.0)	0 (0.0)
HPV33	0 (0.0)	0 (0.0)
HPV35	0 (0.0)	0 (0.0)
HPV51	1 (5.6)	0 (0.0)
HPV58	0 (0.0)	0 (0.0)
HPVx	1 (5.6)	0 (0.0)
Total	18 (100.0)	9 (100.0)

*OPC* oropharyngeal cancer, Others include: Soft palate-C05.1, Uvula-C05.2, Vallecula, Glossoepiglottic fold, laterall and posterior wall of the oropharynx, overlapping lesion of the oropharynx and oropharynx unspecified-C10, Waldeyer ring-C14.2.

**Figure 1:**
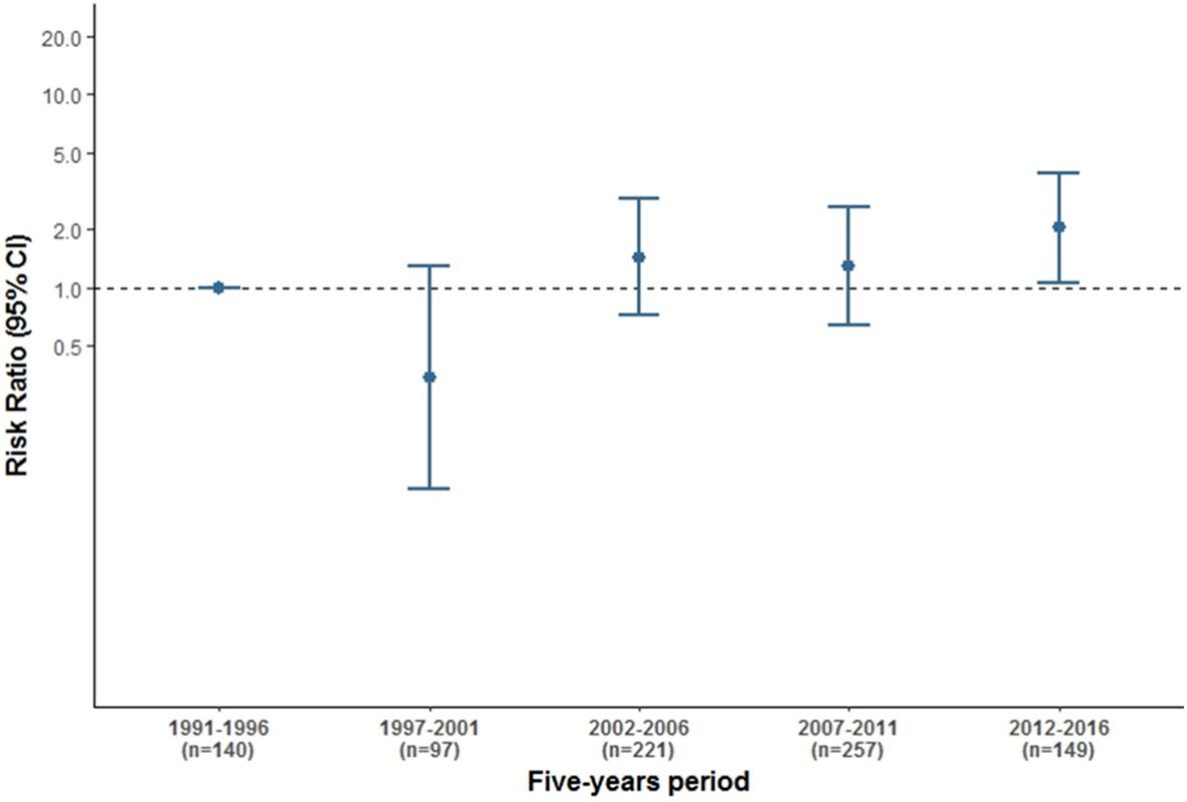
Time trends of relative risk for HPV positivity in OPC. *CI* credibility interval; *OPC* oropharyngeal cancer. Time trends of relative risk for HPV positivity in OPC for each five years period with respect to reference one: 1991–1996. Trends models adjusted by age at diagnosis, gender, period of diagnosis, subsite, stage and tobacco and alcohol consumption. Y axis is represented in logarithmic scale.

Some differences were observed when only p16^INK4a^-positivity was analysed, for all OPC sites and by subsite (supplementary Tables 1 and 2, respectively). Of note, calendar period and age did not show a clear trend in the multivariable analysis for all OPC sites and for tonsil.

### HPV type distribution

Most HPV-DNA positive cases were HPV16 (82.2%), followed by HPV33 (5.9%) and HPV35 (4.0%), (Table 3). When we focused on patients that were double positive for HPV-DNA/p16^INK4a^, the percentage of HPV16 and HPV35 positive patients increased to 88.5% and 5.1%, respectively, but decreased for other genotypes (Table 3). The same was observed specifically for all anatomical subsites, and the highest proportion of HPV16 was found in sites other than tonsil and base of tongue.

### Time trends for HPV positivity

**Figure 2:**
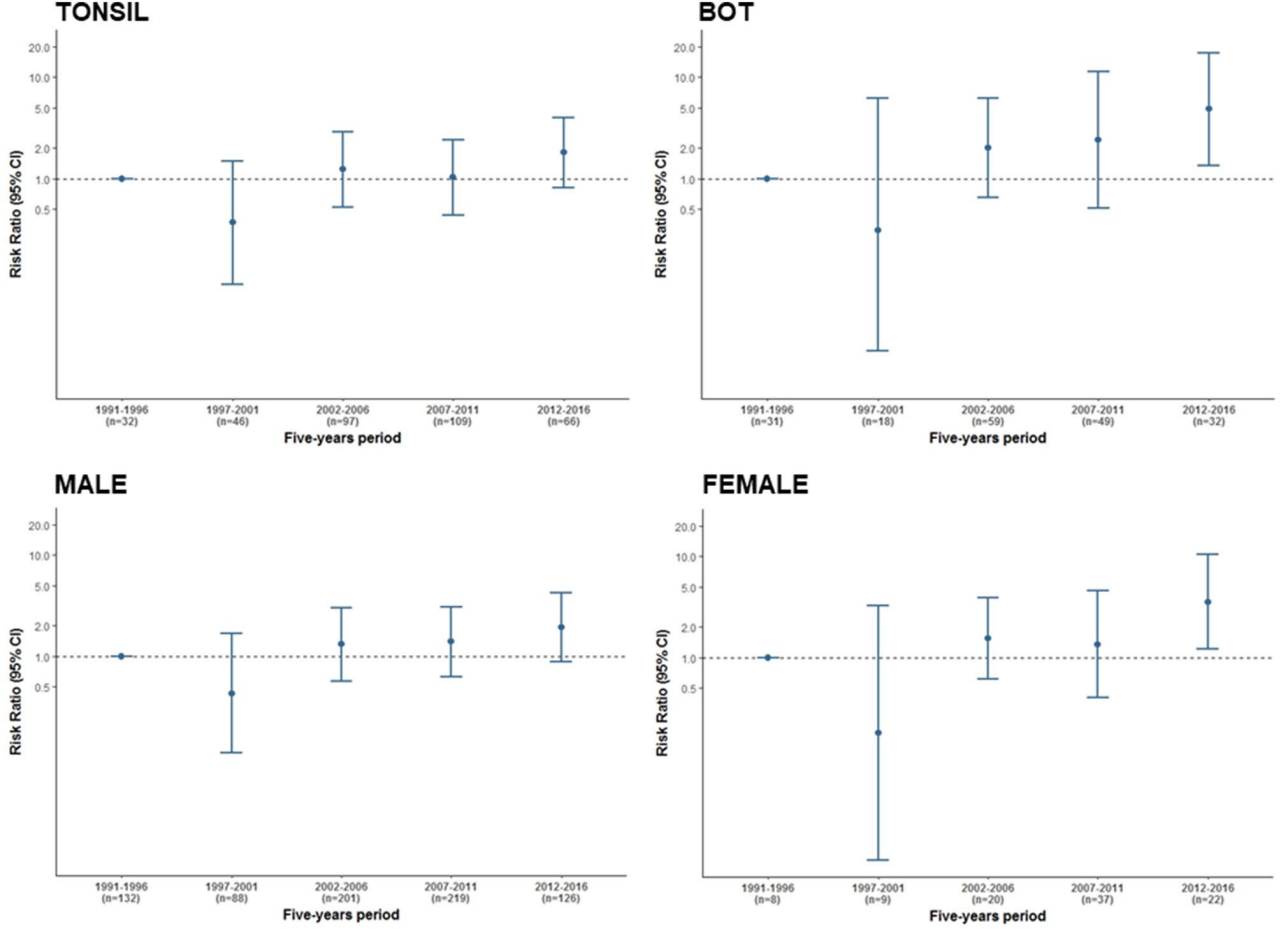
Time trends of relative risk for HPV positivity in OPC by anatomical subsite and gender. *CI* credibility interval, *BOT* base of tongue. Time trends of relative risk for HPV positivity in OPC for each five years period with respect to reference one: 1991–1996. Trends models adjusted by age at diagnosis, gender, period of diagnosis, subsite, stage and tobacco and alcohol consumption. Y axis is represented in logarithmic scale.

In the time-trend analysis, an increasing RR for HPV positivity was observed when period of diagnosis was analysed as a continuous variable (30% for every 5-year period, supplementary Table 5). When period of diagnosis was considered as a categorical variable, we found the increase to be highest (RR = 2.0, 95% CI 1.1–3.9) and with a clear increasing trend only in the last 5-year period (2012–2016, Fig. 1 and supplementary Table 6). Since the most recent patients came mostly from Sant Pau Hospital, a sensitivity analysis including only Sant Pau patients was performed, and equivalent results were obtained when considering period of diagnosis as a continuous variable (RR = 1.2, 95% CI 1.0–1.4). This trend was not observed when period of diagnosis was considered as a categorical variable (RR = 1.8, 95% CI 0.6–5.8), probably due to the lower number of cases. An increasing trend for risk, of 31% and 66% for every 5-year period when period of diagnosis was considered as a continuous variable, was also detected specifically for tonsillar and base of tongue cancers, respectively (supplementary Tables 6 and 7). When period of diagnosis was considered as a categorical variable, the increase was found only for base of tongue patients in the last 5-year period (Fig. 2). Increasing trends in HPV prevalence seemed to be higher in men than in women (Fig. 2 and supplementary Tables 8 and 9).

## Discussion

To our knowledge, this study is the largest and most comprehensive assessment of the epidemiological landscape of HPV-related and non-related OPC in southern Europe. We had previously hypothesized that differences on the epidemiology of HPV-related OPC between regions may reflect distinct trends in temporal, geographical, and sociodemographic shifts in population exposure to both tobacco smoking and oral HPV infection, leading to a rapidly evolving epidemiology of HPV-related OPC ^10^.

Observed HPV prevalences were about 10%, as defined by the percentage of cases that were double positive for HPV-DNA/p16^INK4a^. A recent Italian study also evaluated the role of HPV in patients with newly diagnosed OPC during the period 2000–2018, reporting a prevalence of HPV-driven OPC of 32.3% and a higher prevalence in the most recent years^14^. The lower prevalence observed in our cohort could be explained by the higher number of patients and the inclusion of patients diagnosed at earlier calendar periods than in the Italian study. Moreover, epidemiological differences between the two populations could also exist and account for such differences. As expected^10^, the highest HPV prevalences were observed in tonsillar cancers, with 14% of patients estimated to be HPV-related compared with 8% of base of tongue and 5% of other cancer subsites. Also, in accordance with the literature^8,9^, higher HPV prevalences were observed in more advanced stages based on 7th TNM definition (III and IVa) compared with early stages (I and II).

Our results indicate that HPV prevalences in OPC have started to increase sharply in the most recent years in our setting. However, our results reporting HPV prevalence estimates are based on primary OPC tumours from four hospital-based registries and do not necessarily imply an increasing incidence of HPV-associated OPC in our region. Nevertheless, a previous study estimating the incidence trends of OPC in Spain by using data from population-based registries predicted statistically significant increasing trends for OPC over the period 2003–2017^21^. These results, together with our own, support the hypothesis that these increasing trends for OPC are caused by HPV infection. Of note, age-standardized incidence rates for OPC were estimated in 2018 at 1.4 in Spain and 1.3 in southern Europe, respectively^22^.

If this hypothesis is correct, HPV-related OPC has started to increase in our setting about 20 years later than in other countries such as the US^3^. This two-decade gap is in agreement with the calendar period differences between smoking drops in the US and Spain (supplementary Fig. 2).

However, it is still unclear whether tobacco and/or alcohol use can act as co-factors and/or effect modifiers in the risk of developing HPV-related OPC^23^. Therefore, we cannot rule out the possibility that smoking is associated with an increased risk of HPV-positive OPC, as observed in previous studies in the US^24^.

The increased trends in HPV prevalence were not observed for cancers located at sites other than the tonsil and base of tongue and were more marked in the latter, although the credibility intervals were wider due to the lower number of base of tongue cancers.

After adjustment for confounders, HPV prevalences were found to be higher in younger patients, as observed in other high-burden regions. Of note, most recent publications from the US indicate that a rising proportion of older OPC patients have HPV-positive tumours^25,26^. HPV prevalences were substantially higher in women than in men, in contrast with what is observed, for instance, in the US^3^. This was already observed for Europe in an international cross-sectional study conducted by our group^10^. However, after adjustment for confounders, those gender differences disappeared. A recent Italian study that did not adjust for confounders also observed higher HPV prevalences in women than in men in the univariate analyses^14^. Our results after adjustment for confounders were supported by the finding of a lower number of ever-smokers among female (62/94, 66.0%) than male (650/709, 91.7%) patients. A systematic review on geographical differences in the proportion of HPV prevalence in OPC between men and women revealed that those differences were mainly a consequence of the vast international variation in male smoking habits^27^. Notwithstanding, a population-based study describing time trends of cancer incidence and mortality in Catalonia during the period 1993–2007 detected a rising trend in oral cavity and pharyngeal cancers among women but not among men, presumably explained by a higher and earlier rate of smoking cessation among men than among women^28^. In addition to smoking differences between men and women, HPV transmission appears higher from women to men among heterosexual partners^29^ and differences in sexual behaviours between geographical regions have not been analysed at this level of detail. Intriguingly, although this study was not powered to precisely evaluate this issue due to the low number of female patients, increasing trends in HPV prevalence were suggested to be higher in women than in men (Fig. 2 and Supplementary Tables 8 and 9).

The gender and calendar differences in OPC HPV prevalence between high-burden regions and our setting could also be partially explained by differences in sexual behaviour, which is a clear risk factor for oral HPV acquisition and HPV-related OPC. Indeed, sexual behaviour greatly varies across regions with proportions of ever having oral sex in the US being higher than 65% compared with less than 20% in countries in southern Europe such as Spain^30^. Nevertheless, the CLEOPATRE study, which aimed to estimate the prevalence of cervical HPV infection in Spanish women attending cervical cancer screening, found a higher number of sexual partners and earlier ages for sexual debut in younger (18–25 years) versus the oldest women (56–65 years)^31^. The CLEOPATRE results could thus also partially account for the recent increasing trends in HPV prevalence observed in the present study if it is assumed that changes in oral sex practices have also taken place.

Different trends in tonsillectomy rates could also have contributed to the observed geographical heterogeneity. A previous study from Denmark reported a decrease in tonsillectomy rates concomitant to a simultaneous increase in the risk of OPC^32^. However, other studies did not find that the observed significant increases in OPC were related to declines in tonsillectomies^33,34^ reinforcing increased oral HPV exposure as the likely cause.

The differences in the role of HPV in OPC between anatomical subsites observed in our study are consistent with the results of a recent systematic review^35^ concluding that HPV prevalence differs markedly between OPC subsites and that its role in sites “other” than tonsil or base of tongue is uncertain and needs further evaluation.

A total of 30 out of 108 (27.7%) p16^INK4a^ positive cases were HPV-DNA negative, representing 3.5% of the total sample, despite definition of p16^INK4a^ positivity as staining above > 70%. This percentage is higher than the 10%-20% observed in most studies irrespective of the method of HPV-specific testing applied^36^, although it is lower than the 47% reported in an Italian study that considered a case to be p16^INK4a^ positive if strong nuclear and cytoplasmic staining was present in > 50% of the tumour cells^37^. Classification and potential management of p16^INK4a^-positive—HPV-negative cases is still controversial^38,39^ although we^13^ and others^37,39^ have shown that these cases display similar survival to p16^INK4a^-negative cases. Moreover, molecular characterization of p16^INK4a^-positive—HPV-negative OPC cases has shown a similar genetic profile to HPV-negative tumours^40^. In this regard, we have observed epidemiological differences between OPC cases that were double positive for HPV-DNA/p16^INK4a^ and cases that were p16^INK4a^-positive only, such as the loss of association with age and calendar period. Age discrepancies could be explained by a higher accumulation of mutations with age in the case of p16^INK4a^—positive—HPV-negative patients. A powered study aiming to perform an in-depth comparison between p16^INK4a^-positive—HPV-positive cases and p16^INK4a^-positive—HPV-negative cases is warranted to elucidate these questions.

Our study has several limitations. Selection of patients was not population-based and thus our results cannot be extrapolated to the general population. Moreover, the modest sample size does not allow firm conclusions to be drawn regarding associations with HPV positivity by subsite or gender, for instance. However, according to preliminary estimates of the OPC incidence in Catalonia using data from population-based registries^41^, about one third of OPC cases diagnosed each year in Catalonia are seen at the four hospitals of the study, and our sample represents approximately 16% of all incident OPC cases in the region. The retrospective nature of our cohort may have hampered thorough characterization of patients according to risk factors such as tobacco or alcohol use, since this kind of information could only be partially obtained from medical records. The more recent patients were only available for inclusion in the study for Sant Pau Hospital. However, when we addressed this limitation by performing a sensitivity analysis only considering Sant Pau patients, we obtained equivalent results. In addition, paraffin blocks were not available at diagnosis for a substantial number of patients, notably those with base of tongue carcinoma, a location particularly difficult to biopsy, as well as for patients from older periods. Of note, a fraction of these patients (3% from Bellvitge Hospital) had fine needle aspiration samples. However, these were not included according to our exclusion criteria. Our classification of subsites other than tonsil or base of tongue comprised many different locations, including oropharynx unspecified or overlapping lesions that could also include tonsil and base of tongue.

Our findings indicate that HPV prevalence in OPC has started to sharply increase in the most recent years in our setting. These results, together with previous estimates of increasing trends of OPC in Spain, suggest that HPV-related OPC has started to increase as occurred two decades ago in areas where most OPC cases are currently HPV-related. However, there are some differences between the epidemiology of the disease in our setting and other high-burden regions and we do not yet know whether they may change in the near future to approach the epidemiology of HPV-related OPC in high-burden regions.

Importantly, our results strongly suggest that current estimates of HPV prevalence in southern Europe (probably other regions beyond high-burden areas) are outdated and warrant updated studies in selected populations. Continuing surveillance of sexual behaviours, alongside HPV vaccination status, is warranted to predict future HPV-related OPC burden.

## Supplementary information

Supplementary information
