# Supplementary material for: Epidemiology of human papillomavirus-related oropharyngeal cancer in a classically low-burden region of southern Europe
Source: Sci Rep. 2020 Aug 6;10:13219. doi: 10.1038/s41598-020-70118-7 (PMC7411067; doi:10.1038/s41598-020-70118-7)
Supplement: Supplementary file 1 — Supplementary information [file 41598_2020_70118_MOESM1_ESM.docx]

**SUPPLEMENTARY MATERIAL**

**EPIDEMIOLOGY OF HUMAN PAPILLOMAVIRUS-RELATED OROPHARYNGEAL CANCER IN A CLASSICALLY LOW-BURDEN REGION OF SOUTHERN EUROPE**

Mena M^1,2^*, Frias-Gomez J^1,2,3^*, Taberna M^1,2,3,4,5^, Quirós B^1,2^, Marquez S^1,2^, Clavero O^1,2^, Baena A^6,7^, Lloveras B^8^, Alejo M^9^, León X^10,11^, García J^10,11^, Mesía R^12^, Bermejo O^13^, Bonfill T^14^, Aguila A^15^, Guix M^16^, Hijano R^17^, Pavon MA^1,2^, Torres M^1,2^, Tous S^1,2^, Clèries R^18,19^, Alemany L^1,2,20^

*the authors contributed equally to this work as co-first authors

1. Cancer Epidemiology Research Program, Institut Català d’Oncologia (ICO), L’Hospitalet de Llobregat, Barcelona, Spain
2. Epidemiology, Public Health, Cancer Prevention and Palliative Care Program, Institut d’Investigació Biomèdica de Bellvitge (IDIBELL), L’Hospitalet de Llobregat, Barcelona, Spain
3. University of Barcelona, Barcelona, Spain
4. Department of Medical Oncology, ICO, L’Hospitalet de Llobregat, Barcelona, Spain
5. Program of Molecular Mechanisms and Experimental Therapy in Oncology (ONCOBELL), IDIBELL, L’Hospitalet de Llobregat, Barcelona, Spain
6. Tobacco Control Unit, WHO Collaborating Centre for Tobacco Control, ICO, IDIBELL. L’Hospitalet de Llobregat, Barcelona, Spain
7. Department of e-Health, Faculty of Health Sciences, Universitat Oberta de Catalunya, Barcelona, Spain.
8. Department of Pathology, Hospital del Mar, Barcelona, Spain
9. Department of Pathology, Hospital General de l’Hospitalet, Barcelona, Spain
10. Department of Otorhinolaryngology. Hospital de la Santa Creu i Sant Pau. Barcelona, Spain
11. Centro de Investigación Biomédica en Red de Bioingeniería, Biomateriales y Nanomedicina (CIBER-BBN). Instituto de Salud Carlos III, Madrid, Spain
12. Department of Medical Oncology, ICO, B-ARGO group, Badalona, Barcelona, Spain
13. Department of Plastic Surgery, Hospital Universitari de Bellvitge, L’Hospitalet de Llobregat, Barcelona, Spain
14. Department of Medical Oncology, Hospital Universitari Parc Taulí. Sabadell, Barcelona, Spain
15. Department of Otorhinolaryngology, Hospital Universitari Parc Taulí. Sabadell, Barcelona, Spain
16. Department of Medical Oncology, Hospital del Mar, Barcelona, Spain
17. Department of Otorhinolaryngology, Hospital del Mar, Barcelona, Spain
18. Pla Director d'Oncologia, ICO, IDIBELL. L’Hospitalet de Llobregat, Barcelona, Spain
19. Dept. Ciències Clíniques, Universitat de Barcelona, Barcelona, Spain
20. Centro de Investigación Biomédica en Red: Epidemiología y Salud Pública (CIBERESP), Instituto de Salud Carlos III, Madrid, Spain

**CORRESPONDING AUTHOR:** Dr Laia Alemany / Marisa Mena

Cancer Epidemiology Research Program, ICO, IDIBELL. Address: Av. Gran Via de L'Hospitalet 199-203, 08908 L'Hospitalet de Llobregat. Barcelona, Spain; Phone: (+34) 93 2607812; E-mail: [lalemany@iconcologia.net / mmena@iconcologia.net](mailto:lalemany@iconcologia.net%20/%20mmena@iconcologia.net)

**Supplementary Table 1.** **Association of demographics and clinical characteristics of OPC patients and p16^INK4a^ positivity.**

| **Characteristics** | **OPC samples**  **(n =861)**  **No. (%)**^a^ | **p16^INK4a^ high expression (n =108)** | | | |
| --- | --- | --- | --- | --- | --- |
|  |  | **Crude Prevalence**  **No. (%)^b^** | **Adjusted**  **Prevalence^c^**  **(%)** | **Crude OR**  **[95%CI]** | **Adjusted OR^c^**  **[95%CI]** |
| **Age at diagnosis**  ≤ 60  > 60  Mean age at diagnosis (SD)  Age range | 456 (53.1)  403 (46.9)  60.3 (10.6)  28-93 | 56 (12.3)  51 (12.7)  60.3 (13.2)  28-93 | 14.9  11.6 | 1.0 [0.6-1.4]  Ref. | 1.5 [0.9-2.3]  Ref. |
| **Gender**  Male  Female | 764 (88.9)  95 (11.1) | 81 (10.6)  27 (28.4) | 12.6  16.1 | Ref.  **3.3 [2.0-5.4]** | Ref.  1.4 [0.8-2.6] |
| **Center^d^**  H Mar  H ICO-Bellvitge  H Parc Taulí  H Sant Pau | 99 (11.5)  240 (27.9)  84 (9.8)  438 (50.9) | 9 (9.1)  24 (10.0)  10 (11.9)  65 (14.8) |  | Ref.  1.1 [0.5-2.3]  1.3 [0.5-3.2]  1.7 [0.8-3.4] |  |
| **Period of diagnosis**  1991-1996  1997-2001  2002-2006  2007-2011  2012-2016 | 140 (16.3)  97 (11.3)  221 (25.7)  256 (29.7)  147 (17.1) | 13 (9.3)  10 (10.3)  22 (10.0)  28 (10.9)  35 (23.8) | 13.4  11.9  11.2  10.8  20.7 | Ref.  1.1 [0.5-2.5]  1.0 [0.5-2.1]  1.2 [0.6-2.2]  **2.9 [1.5-5.6]** | Ref.  0.9 [0.3-2.1]  0.8 [0.4-1.7]  0.7 [0.4-1.6]  1.9 [0.9-4.1] |
| **Tobacco use**  Non smoker  < 20 cigarettes/day  ≥ 20 cigarettes/day | 88 (11.0)  116 (14.5)  597 (74.5) | 38 (43.2)  22 (19.0)  46 (7.7) | 27.0  15.0  9.5 | **8.8 [5.3-14.7]**  **2.7 [1.6-4.7]**  Ref. | **4.3 [2.2-8.3]**  1.8 [0.9-3.3]  Ref. |
| **Alcohol consumption**  Non drinker  < 100 grams/day   ≥ 100 grams/day | 160 (19.9)  243 (30.2)  401 (49.9) | 52 (32.5)  35 (14.4)  19 (4.7) | 20.5  15.5  6.8 | **9.2 [5.3-16.0]**  **3.2 [1.8-5.7]**  Ref. | **4.2 [2.1-8.4]**  **2.8 [1.5-5.2]**  Ref. |
| **Subsite**  Tonsil  BOT  Tonsil & BOT  Others^e^ | 348 (40.4)  188 (21.8)  19 (2.2)  306 (35.5) | 68 (19.5)  20 (10.6)  1 (5.3)  19 (6.2) | 18.7  9.6  7.8  8.5 | **3.6 [2.1-6.0]**  1.7 [0.9-3.3]  0.9 [0.1-5.1]  Ref. | **3.0 [1.7-5.3]**  1.2 [0.6-2.4]  0.9 [0.1-5.6]  Ref. |
| **Stage (7^th^ edition TNM)**  I&II  III  IVa  IVb  IVc | 182 (21.2)  184 (21.4)  391 (45.6)  82 (9.6)  19 (2.2) | 12 (6.6)  25 (13.6)  62 (15.9)  6 (7.3)  3 (15.8) | 6.7  14.0  16.2  9.2  20.9 | **Ref.**  **2.1 [1.1-4.1]**  **2.5 [1.4-4.6]**  1.0 [0.4-2.7]  2.3 [0.6-8.4] | Ref.  **2.7 [1.2-5.9]**  **3.3 [1.6-6.7]**  1.5 [0.5-4.4]  **4.9 [1.1-21.8]** |
| **Histology^d^**  SCC Conventional keratinizing  SCC Conventional non keratinizing  SCC Basaloid, papillary, exophitic  SCC Sarcomatoid  Non SCC^f^ | 547 (63.5)  232 (26.9)  73 (8.5)  3 (0.3)  6 (0.7) | 34 (6.2)  39 (16.8)  33 (45.2)  0 (0.0)  2 (33.3) |  | Ref.  **2.9 [1.8-4.7]**  **11.8 [6.7-20.9]**  0.6 [0.0-19.0]  **5.6 [1.1-30.0]** |  |
| **TOTAL** | 861 | 108 (12.5) | 13.3 |  |  |

OPC: Oropharyngeal carcinoma; SD: Standard deviation; H: Hospital; SCC: Squamous cell carcinoma; BOT: Base of the tongue; CI: Credibility interval; ^a^Column percentage. ^b^Row percentage. ^c^Adjusted by age, gender, period of diagnosis, subsite, tobacco and alcohol consumption and stage. ^d^Not considered in the multivariable model. ^e^Others include: Soft palate-C05.1, Uvula-C05.2, Vallecula, Glossoepiglottic fold, lateral and posterior wall of the oropharynx, overlapping lesion of the oropharynx and oropharynx unspecified-C10, Waldeyer ring-C14.2. ^f^Non SCC include: 4 undifferentiated (2 of them p16^INK4a^ positive) and 3 neuroendocrine carcinomas. In bold those estimates showing a clear association with p16^INK4a^ positivity (i.e. credibility intervals do not contain 1.0).

**Supplementary Table 2.** **Association of demographics and clinical characteristics of OPC patients and p16^INK4a^ positivity stratified by the three major anatomical sites (Tonsil / Base of the Tongue / Others).**

| **Characteristics** | **p16^INK4a^ high expression** | | | | | | | | |
| --- | --- | --- | --- | --- | --- | --- | --- | --- | --- |
|  | **Tonsil samples (n=348)**  **Prevalence n/N (%)** | **Crude OR**  **[95%CI]** | **Adjusted OR^a^**  **[95%CI]** | **BOT samples**  **(n =188)**  **Prevalence n/N (%)** | **Crude OR**  **[95%CI]** | **Adjusted OR^a^**  **[95%CI]** | **Others**  **samples^b^**  **(n =306)**  **Prevalence n/N (%)** | **Crude OR**  **[95%CI]** | **Adjusted OR^a^**  **[95%CI]** |
| **Age at diagnosis**  ≤ 60  > 60 | 34/182 (18.7)  34/166 (20.5) | 0.9 [0.5-1.5]  Ref. | 1.7 [0.9-3.3]  Ref. | 12/100 (12.0)  7/87 (8.0) | 1.5 [0.6-3.9]  Ref. | 1.3 [0.4-4.4] Ref. | 9/163 (5.5)  10/142 (7.0) | 0.8 [0.3-1.9]  Ref. | 0.7 [0.3-2.0]  Ref. |
| **Gender**  Male  Female | 53/301 (17.6)  15/46 (32.6) | Ref.  **2.2 [1.1-4.3]** | Ref.  0.9 [0.4-2.2] | 14/167 (8.4)  6/21 (28.6) | Ref.  **3.9 [1.4-11.4]** | Ref.  1.7 [0.4-7.3] | 14/279 (5.0)  5/26 (19.2) | Ref.  **4.0 [1.4-12.0]** | Ref.  2.7 [0.8-9.9] |
| **Center^c^**  H Mar  H ICO-Bellvitge  H Parc Taulí  H Sant Pau | 8/49 (16.3)  18/107 (16.8)  7/39 (17.9)  35/153 (22.9) | Ref.  1.0 [0.4-2.4]  1.1 [0.4-3.1]  1.5 [0.7-3.3] |  | 1/21 (4.8)  3/70 (4.3)  0/13 (0.0)  16/84 (19.0) | Ref.  0.9 [0.1-5.1]  0.3 [0.0-7.6]  4.3 [0.9-21.9] |  | 0/27 (0.0)  2/51 (3.9)  3/27 (11.1)  14/201 (7.0) | Ref.  1.5 [0.2-11.1]  4.3 [0.6-30.8]  2.8 [0.5-16.4] |  |
| **Period of diagnosis**  1991-1996  1997-2001  2002-2006  2007-2011  2012-2016 | 4/32 (12.5)  9/46 (19.6)  15/97 (15.5)  18/109 (16.5)  22/64 (34.4) | Ref.  1.5 [0.5-4.4]  1.1 [0.4-3.1]  1.2 [0.4-3.3]  **3.1 [1.2-8.5]** | Ref.  1.6 [0.5-5.6]  1.3 [0.4-4.1]  1.0 [0.3-3.2]  3.2 [0.9-10.5] | 2/31 (6.5)  1 /18 (5.6)  4/59 (6.8)  3/48 (6.3)  10/32 (31.3) | Ref.  0.8 [0.1-5.6]  0.9 [0.2-3.9]  0.9 [0.2-3.9]  **5.5 [1.5-20.7]** | Ref.  1.2 [0.1-10.1]  0.9 [0.2-4.5]  1.2 [0.2-6.3]  **5.3 [1.1-24.7]** | 7/76 (9.2)  0/31 (0.0)  3/57 (5.3)  6/92 (6.5)  3/50 (6.0) | Ref.  0.1 [0.0-2.1]  0.6 [0.2-2.3]  0.8 [0.3-2.3]  0.7 [0.2-2.6] | Ref.  0.1 [0.0-2.3]  0.5 [0.1-2.2]  0.4 [0.1-1.3]  0.5 [0.1-1.9] |
| **Tobacco use**  Non smoker  < 20 cigarettes/day  ≥ 20 cigarettes/day | 26/45 (57.8)  15/54 (27.8)  26/234 (11.1) | **10.3 [5.1-20.8]**  **2.9 [1.4-5.9]**  Ref. | **7.4 [3.1-17.9]**  1.7 [0.8-3.9]  Ref. | 8/20 (40.0)  5/18 (27.8)  6/135 (4.4) | **11.6 [3.7-36.6]**  **6.6 [1.9-22.8]**  Ref. | 3.6 [0.8-15.5]  **4.8 [1.1-20.7]**  Ref. | 4/22 (18.2)  2/40 (5.0)  13/214 (6.1) | 3.1 [0.9-10.1]  0.8 [0.2-3.3]  Ref. | 1.3 [0.3-6.1]  0.5 [0.1-2.4]  Ref. |
| **Alcohol consumption**  Non drinker  < 100 grams/day   ≥ 100 grams/day | 35/90 (38.9)  24/94 (25.5)  8/151 (5.3) | **10.1 [4.6-22.2]**  **5.4 [2.4-12.2]**  Ref. | **5.8 [2.3-14.8]**  **4.8 [2.0-11.2]**  Ref. | 11/31 (35.5)  5/57 (8.8)  3/87 (3.4) | **12.0 [3.6-40.6]**  2.1 [0.6-7.8]  Ref. | 4.7 [0.9-22.7]  2.0 [0.5-8.3]  Ref. | 6/37 (16.2)  6/85 (7.1)  7/154 (4.5) | **3.6 [1.2-10.8]**  1.5 [0.5-4.2]  Ref. | 3.5 [0.8-15.6]  1.8 [0.6-5.5]  Ref. |
| **Stage (7^th^ edition TNM)**  I&II  III  IVa  IVb  IVc | 9/88 (10.2)  18/72 (25.0)  35/151 (23.2)  5/27 (18.5)  1/7 (14.3) | Ref.  **2.6 [1.2-5.9]**  **2.4 [1.2-5.0]**  1.8 [0.6-5.4]  1.2 [0.2-8.6] | Ref.  **3.9 [1.5-10.3]**  **2.9 [1.2-6.7]**  1.9 [0.5-6.6]  3.0 [0.3-29.0] | 1/17 (5.9)  4/38 (10.5)  15/99 (15.2)  0/28 (0.0)  0/6 (0.0) | 0.7 [0.1-4.5]  Ref.  1.7 [0.6-5.3]  0.1 [0.0-2.1]  0.4 [0.0-8.0] | 0.4 [0.0-8.3]  Ref.  2.7 [0.6-11.7]  0.2 [0.0-4.1]  0.4 [0.0-12.0] | 1/74 (1.4)  3/72 (4.2)  12/130 (9.2)  1/24 (4.2)  2/6 (33.3) | 0.4 [0.1-2.2]  Ref.  2.1 [0.7-6.4]  0.9 [0.1-6.2]  **7.8 [1.2-51.3]** | 0.5 [0.1-3.2]  Ref.  2.1 [0.7-6.8]  1.1 [0.2-7.5]  **16.2 [1.7-149.8]** |
| **Histology^c^**  SCC Conventional keratinizing  SCC Conventional non keratinizing  SCC Basaloid, papillary, exophitic  SCC Sarcomatoid  Non SCC^d^ | 24/221 (10.9)  20/87 (23.0)  23/36 (63.9)  0/1 (0.0)  1/3 (33.3) | Ref.  **2.3 [1.2-4.4]**  **13.4 [6.1-29.2]**  0.7 [0.0-29.3]  2.7 [0.3-24.5] |  | 4/114 (3.5)  8/54 (14.8)  7/16 (43.8)  0/1 (0.0)  1/3 (33.3) | Ref.  **3.6 [1.2-11.0]**  **15.3 [4.3-54.8]**  0.8 [0.0-54.0]  6.3 [0.6-70.8] |  | 6/199 (3.0)  11/87 (12.6)  2/19 (10.5)  0/1 (0.0)  - | Ref.  **4.1 [1.6-10.7]**  2.9 [0.6-14.3]  0.9 [0.0-62.8]  - |  |
| **TOTAL** | 68/348 (19.5) |  |  | 20/188 (10.6) |  |  | 19/306 (6.2) |  |  |

OPC: Oropharyngeal carcinoma; SD: Standard deviation; H: Hospital; SCC: Squamous cell carcinoma; BOT: Base of the tongue; CI: Credibility interval; ^a^Adjusted by age, gender, period of diagnosis, tobacco and alcohol consumption and stage. ^b^Others include: Soft palate-C05.1, Uvula-C05.2, Vallecula, Glossoepiglottic fold, lateral and posterior wall of the oropharynx, overlapping lesion of the oropharynx and oropharynx unspecified-C10, Waldeyer ring-C14.2. ^c^Not considered in the multivariable model. ^f^Non SCC include: 4 undifferentiated (2 of them p16^INK4a^ positive) and 3 neuroendocrine carcinomas. In bold those estimates showing a clear association with p16^INK4a^ positivity (i.e. credibility intervals do not contain 1.0).

**Supplementary Table 3. Crude and adjusted prevalence of HPV-related OPC patients (as defined by double positivity for HPV-DNA/p16^INK4a^) by demographics and clinical characteristics, stratified by the three major anatomical sites (Tonsil / Base of the Tongue / Others).**

| **Characteristics** | **HPV-DNA detection AND p16^INK4a^ high expression** | | | | | | | | |
| --- | --- | --- | --- | --- | --- | --- | --- | --- | --- |
|  | **Tonsil samples (n=350)**  **No. (%)^a^** | **Crude Prevalence**  **No. (%)^b^** | **Adjusted**  **Prevalence^c^**  **(%)** | **BOT samples**  **(n =189)**  **No. (%)^a^** | **Crude Prevalence**  **No. (%)^b^** | **Adjusted**  **Prevalence^c^**  **(%)** | **Others^d^**  **samples**  **(n =306)**  **No. (%)^a^** | **Crude Prevalence**  **No. (%)^b^** | **Adjusted**  **Prevalence^c^**  **(%)** |
| **Age at diagnosis**  ≤ 60  > 60  Mean age at diagnosis (SD)  Age range | 182 (52.0)  168 (48.0)  60.6 (11.2)  28-93 | 30 (16.5)  23 (13.7)  59.2 (14.6)  28-93 | 21.8  11.4 | 100 (53.2)  88 (46.8)  60.2 (10.2)  31-86 | 12 (12.0)  3 (3.4)  55.1 (9.7)  44-83 | 12.1  4.9 | 163 (53.4)  142 (46.6)  60.3 (10.3)  38-87 | 4 (2.5)  5 (3.5)  62.4 (10.6)  50-82 | 3.5  3.1 |
| **Gender**  Male  Female | 302 (86.5)  47 (13.5) | 40 (13.2)  13 (27.7) | 15.6  17.2 | 168 (88.9)  21 (11.1) | 10 (6.0)  6 (28.6) | 8.0  10.8 | 279 (91.5)  26 (8.5) | 7 (2.5)  2 (7.7) | 3.3  3.5 |
| **Center^e^**  H Mar  H ICO-Bellvitge  H Parc Taulí  H Sant Pau | 49 (14.0)  108 (30.9)  39 (11.1)  154 (44.0) | 5 (10.2)  15 (13.9)  5 (12.8)  28 (18.2) | - | 22 (11.6)  70 (37.0)  13 (6.9)  84 (44.4) | 1 (4.6)  3 (4.3)  0 (0.0)  12 (14.3) |  | 27 (8.8)  51 (16.7)  27 (8.8)  201 (65.7) | 0 (0.0)  0 (0.0)  0 (0.0)  9 (4.5) |  |
| **Period of diagnosis**  1991-1996  1997-2001  2002-2006  2007-2011  2012-2016 | 32 (9.1)  46 (13.1)  97 (27.7)  109 (31.1)  66 (18.9) | 4 (12.5)  2 (4.3)  10 (10.3)  15 (13.8)  22 (33.3) | 14.9  5.9  14.0  12.9  30.3 | 31 (16.4)  18 (9.5)  59 (31.2)  49 (25.9)  32 (16.9) | 1 (3.2)  0 (0.0)  4 (6.8)  3 (6.1)  8 (25.0) | 4.4  1.7  6.7  9.2  19.2 | 76 (24.8)  31 (10.1)  57 (18.6)  92 (30.1)  50 (16.3) | 2 (2.6)  0 (0.0)  2 (3.5)  3 (3.3)  2 (4.0) | 3.1  1.0  4.6  3.0  4.1 |
| **Tobacco use**  Non smoker  < 20 cigarettes/day  ≥ 20 cigarettes/day | 46 (13.7)  54 (16.1)  235 (70.1) | 24 (52.2)  14 (25.9)  15 (6.4) | 39.0  17.0  8.7 | 21 (12.1)  18 (10.3)  135 (77.6) | 8 (38.1)  4 (22.2)  4 (3.0) | 14.9  13.1  5.4 | 22 (8.0)  40 (14.5)  214 (77.5) | 4 (18.2)  1 (2.5)  4 (1.9) | 10.6  2.6  2.2 |
| **Alcohol consumption**  Non drinker  <100grams/day   ≥100grams/day | 91 (27.0)  95 (28.2)  151 (44.8) | 31 (34.1)  19 (20.0)  3 (2.0) | 22.0  22.1  4.4 | 31 (17.6)  58 (33.0)  87 (49.4) | 11 (35.5)  4 (6.9)  1 (1.1) | 17.3  7.4  3.3 | 37 (13.4)  85 (30.8)  154 (55.8) | 4 (10.8)  4 (4.7)  1 (0.6) | 5.6  4.6  1.3 |
| **Stage (7^th^ edition TNM)**  I&II  III  IVa  IVb  IVc | 89 (25.7)  72 (20.7)  152 (43.8)  27 (7.8)  7 (2.0) | 6 (6.7)  13 (18.1)  31 (20.4)  3 (11.1)  0 (0.0) | 8.9  22.3  18.4  10.0  6.4 | 17 (9.0)  38 (20.1)  100 (52.9)  28 (14.8)  6 (3.2) | 0 (0.0)  4 (10.5)  12 (12.0)  0 (0.0)  0 (0.0) | 4.8  8.0  10.3  3.0  3.8 | 74 (24.2)  72 (23.5)  130 (42.5)  24 (7.8)  6 (2.0) | 0 (0.0)  2 (2.8)  6 (4.6)  1 (4.2)  0 (0.0) | 0.5  2.9  4.8  5.5  2.2 |
| **Histology^e^**  SCC Conventional keratinizing  SCC Conventional non keratinizing  SCC Basaloid, papillary, exophitic  SCC Sarcomatoid  Non SCC^f^ | 221 (63.1)  88 (25.1)  36 (10.3)  1 (0.3)  4 (1.1) | 15 (6.8)  18 (20.5)  20 (55.6)  0 (0.0)  0 (0.0) | - | 114 (60.3)  55 (29.1)  16 (8.5)  1 (0.5)  3 (1.6) | 3 (2.6)  5 (9.1)  7 (43.8)  0 (0.0)  1 (33.3) |  | 199 (65.0)  87 (28.4)  19 (6.2)  1 (0.3)  - | 2 (1.0)  5 (5.7)  2 (10.5)  0 (0.0)  - |  |
| **TOTAL** | 350 | 53 (15.1) | 16.0 | 189 | 16 (8.5) | 8.8 | 306 | 9 (2.9) | 3.3 |

OPC: Oropharyngeal carcinoma; SD: Standard deviation; H: Hospital; SCC: Squamous cell carcinoma; BOT: Base of the tongue; CI: Credibility interval; ^a^Column percentage. ^b^Row Percentage. ^c^Adjusted by age, gender, period of diagnosis, tobacco and alcohol consumption and stage. ^d^Others include: Soft palate-C05.1, Uvula-C05.2, Vallecula, Glossoepiglottic fold, lateral and posterior wall of the oropharynx, overlapping lesion of the oropharynx and oropharynx unspecified-C10, Waldeyer ring-C14.2. ^e^Not considered in the multivariable model. ^f^Non SCC include: 4 undifferentiated (2 p16^INK4a^ positive) and 3 neuroendocrine carcinomas.

**Supplementary Table 4. Crude and adjusted prevalence of HPV-related OPC patients (as defined by p16^INK4a^ positivity) by demographics and clinical characteristics, stratified by the three major anatomical sites (Tonsil / Base of the Tongue / Others).**

| **Characteristics** | **p16^INK4a^ high expression** | | | | | | | | |
| --- | --- | --- | --- | --- | --- | --- | --- | --- | --- |
|  | **Tonsil samples (n=348)**  **No. (%)^a^** | **Crude Prevalence**  **No. (%)^b^** | **Adjusted**  **Prevalence^c^**  **(%)** | **BOT samples**  **(n =188)**  **No. (%)^a^** | **Crude Prevalence**  **No. (%)^b^** | **Adjusted**  **Prevalence^c^**  **(%)** | **Others^d^**  **samples**  **(n =306)**  **No. (%)^a^** | **Crude Prevalence**  **No. (%)^b^** | **Adjusted**  **Prevalence^c^**  **(%)** |
| **Age at diagnosis**  ≤ 60  > 60  Mean age at diagnosis (SD)  Age range | 182 (52.3)  166 (47.7)  60.5 (11.1)  28-93 | 34 (18.7)  34 (20.5)  61.0 (14.4)  28-93 | 23.5  17.5 | 100 (53.5)  87 (46.5)  60.1 (10.2)  31-86 | 12 (12.0)  7 (8.0)  59.1 (11.9)  44-83 | 11.4  9.5 | 163 (53.4)  142 (46.6)  60.3 (10.3)  38-87 | 9 (5.5)  10 (7.0)  59.6 (9.6)  40-82 | 8.0  6.1 |
| **Gender**  Male  Female | 301 (87.7)  46 (13.3) | 53 (17.6)  15 (32.6) | 20.5  19.6 | 167 (88.8)  21 (11.2) | 14 (8.4)  6 (28.6) | 9.7  13.7 | 279 (91.5)  26 (8.5) | 14 (5.0)  5 (19.2) | 6.0  14.0 |
| **Center^e^**  H Mar  H ICO-Bellvitge  H Parc Taulí  H Sant Pau | 49 (14.1)  107 (30.7)  39 (11.2)  153 (44.0) | 8 (16.3)  18 (16.8)  7 (17.9)  35 (22.9) |  | 21 (11.2)  70 (37.2)  13 (6.9)  84 (44.7) | 1 (4.8)  3 (4.3)  0 (0.0)  16 (19.0) |  | 27 (8.8)  51 (16.7)  27 (8.8)  201 (65.7) | 0 (0.0)  2 (3.9)  3 (11.1)  14 (7.0) |  |
| **Period of diagnosis**  1991-1996  1997-2001  2002-2006  2007-2011  2012-2016 | 32 (9.2)  46 (13.2)  97 (27.9)  109 (31.3)  64 (18.4) | 4 (12.5)  9 (19.6)  15 (15.5)  18 (16.5)  22 (34.4) | 15.8  21.2  18.4  16.0  31.1 | 31 (16.5)  18 (9.6)  59 (31.4)  48 (25.5)  32 (17.0) | 2 (6.5)  1 (5.6)  4 (6.8)  3 (6.3)  10 (31.3) | 7.5  8.4  7.2  8.8  22.6 | 76 (24.8)  31 (10.1)  57 (18.6)  92 (30.1)  50 (16.4) | 7 (9.2)  0 (0.0)  3 (5.3)  6 (6.5)  3 (6.0) | 11.9  2.1  7.3  5.4  6.3 |
| **Tobacco use**  Non smoker  < 20 cigarettes/day  ≥ 20 cigarettes/day | 45 (13.5)  54 (16.2)  234 (70.3) | 26 (57.8)  15 (27.8)  26 (11.1) | 45.9  20.5  13.7 | 20 (11.6)  18 (10.4)  135 (78.0) | 8 (40.0)  5 (27.8)  6 (4.4) | 16.9  20.3  6.7 | 22 (8.0)  40 (14.5)  214 (77.5) | 4 (18.2)  2 (5.0)  13 (6.1) | 9.1  3.9  7.2 |
| **Alcohol consumption**  Non drinker  < 100 grams/day   ≥ 100 grams/day | 90 (26.9)  94 (28.1)  151 (45.1) | 35 (38.9)  24 (25.5)  8 (5.3) | 29.4  26.1  8.4 | 31 (17.7)  57 (32.6)  87 (49.7) | 11 (35.5)  5 (8.8)  3 (3.4) | 18.0  10.0  5.9 | 37 (13.4)  85 (30.8)  154 (55.8) | 6 (16.2)  6 (7.1)  7 (4.5) | 13.9  7.9  4.8 |
| **Stage (7^th^ edition TNM)**  I&II  III  IVa  IVb  IVc | 88 (25.5)  72 (20.9)  151 (43.8)  27 (7.8)  7 (2.0) | 9 (10.2)  18 (25.0)  35 (23.2)  5 (18.5)  1 (14.3) | 11.4  26.8  22.5  17.3  23.3 | 17 (9.0)  38 (20.2)  99 (52.7)  28 (14.9)  6 (3.2) | 1 (5.9)  4 (10.5)  15 (15.2)  0 (0.0)  0 (0.0) | 3.2  7.2  14.5  1.8  3.6 | 74 (24.2)  72 (23.5)  130 (42.5)  24 (7.8)  6 (2.0) | 1(1.4)  3 (4.2)  12 (9.2)  1 (4.2)  2 (33.3) | 2.4  4.7  9.3  5.0  38.9 |
| **Histology^e^**  SCC Conventional keratinizing  SCC Conventional non keratinizing  SCC Basaloid, papillary, exophitic  SCC Sarcomatoid  Non SCC^f^ | 221 (63.5)  87 (25.0)  36 (10.3)  1 (0.3)  3 (0.9) | 24 (10.9)  20 (23.0)  23 (63.9)  0 (0.0)  1 (33.3) |  | 114 (60.6)  54 (28.7)  16 (8.5)  1 (0.5)  3 (1.6) | 4 (3.5)  8 (14.8)  7 (43.8)  0 (0.0)  1 (33.3) |  | 199 (65.0)  87 (28.4)  19 (6.2)  1 (0.3)  - | 6 (3.0)  11 (12.6)  2 (10.5)  0 (0.0)  - |  |
| **TOTAL** | 348 | 68 (19.5) | 20.3 | 188 | 20 (10.6) | 10.6 | 306 | 19 (6.2) | 7.0 |

OPC: Oropharyngeal carcinoma; SD: Standard deviation; H: Hospital; SCC: Squamous cell carcinoma; BOT: Base of the tongue; CI: Credibility interval; ^a^Column percentage. ^b^Row percentage. ^c^Adjusted by age, gender, period of diagnosis, tobacco and alcohol consumption and stage. ^d^Others include: Soft palate-C05.1, Uvula-C05.2, Vallecula, Glossoepiglottic fold, lateral and posterior wall of the oropharynx, overlapping lesion of the oropharynx and oropharynx unspecified-C10, Waldeyer ring-C14.2. ^e^Not considered in the multivariable model. ^f^Non SCC include: 4 undifferentiated (2 of them p16^INK4a^ positive) and 3 neuroendocrine carcinomas.

**Supplementary Table 5. Time trend analysis for HPV positivity (HPV-DNA/p16^INK4a^) in OPC patients, with period as continuous.**

|  | **OPC samples (n=864)** | | | |
| --- | --- | --- | --- | --- |
|  | **No. (%)** | **RR^a^** | **[95% CI]** | **Probability^b^ %** |
| **Age at diagnosis**  ≤ 60  > 60 | 456 (52.9)  406 (47.1) | **1.8**  Ref. | [**1.3-2.6**] | 99.98 |
| **Gender**  Male  Female | 766 (88.9)  96 (11.1) | Ref.  **1.6** | [**1.2-2.1**] | 99.9 |
| **Period of diagnosis**  Five-years | 864 (100.0) | **1.3** | [**1.1-1.5**] | 99.97 |
| **Tobacco use**  Non Smoker  < 20 cigarettes/day  ≥ 20 cigarettes/day | 90 (11.2)  116 (14.4)  598 (74.4) | **3.9**  **2.5**  Ref. | [**2.4-6.5**]  [**1.5-4.1**] | 100  99.98 |
| **Alcohol consumption**  Non Drinker  < 100 grams/day  ≥ 100 grams/day | 161 (20.0)  245 (30.4)  401 (49.7) | **6.5**  **6.3**  Ref. | [**2.8-15.4**]  [**2.7-14.6**] | 99.99  99.99 |
| **Subsite**  Tonsil  BOT  Tonsil&BOT  Others^b^ | 350 (40.5)  189 (21.9)  19 (2.2)  306 (35.4) | **2.8**  **2.0**  0.3  Ref. | [**1.5-5.2**]  [**1.1-4.0**]  [0.0-6.0] | 99.95  97.7  22.0 |
| **Stage (7th edition)**  I&II  III  IVa  IVb  IVc | 183 (21.3)  184 (21.4)  393 (45.6)  82 (9.5)  19 (2.2) | Ref.  **2.7**  **2.5**  1.6  0.4 | [**1.3-5.5**]  [**1.2-5.0**]  [0.6-4.3]  [0.0-8.3] | 99.6  99.5  81.2  27.3 |
| **AIC** | 342.9 | | | |

OPC: Oropharyngeal carcinoma; BOT: Base of the tongue; RR: Relative Risk; CI: Credibility interval. ^a^Adjusted by age, gender, period of diagnosis, subsite, tobacco and alcohol consumption. ^b^Probability of an increasing relative risk. In bold those estimates showing a clear association with HPV-DNA/p16^INK4a^ positivity (i.e. credibility intervals do not contain 1.0).

**Supplementary Table 6. Time trend analysis for HPV positivity (HPV-DNA/p16^INK4a^) in OPC patients, with period as categorical, overall, by anatomical subsite and by gender**

|  | **Sample size** | **RR [95% CI]^a^** |
| --- | --- | --- |
| **OPC** | 864 |  |
| Period of diagnosis  1991-1996  1997-2001  2002-2006  2007-2011  2012-2016 | 140  97  221  257  149 | Ref.  0.3 [0.1-1.3]  1.4 [0.7-2.9]  1.3 [0.6-2.6]  **2.0 [1.1-3.9]** |
| **BOT** | 189 |  |
| Period of diagnosis  1991-1996  1997-2001  2002-2006  2007-2011  2012-2016 | 31  18  59  49  32 | Ref.  0.3 [0.0-6.2]  2.0 [0.7-6.2]  2.4 [0.5-11.2]  **4.8 [1.3-17-4]** |
| **Tonsil** | 350 |  |
| Period of diagnosis  1991-1996  1997-2001  2002-2006  2007-2011  2012-2016 | 32  46  97  109  66 | Ref.  0.4 [0.1-1.5]  1.2 [0.5-2.9]  1.0 [0.4-2.4]  1.8 [0.8-4.0] |
| **Males** | 766 |  |
| Period of diagnosis  1991-1996  1997-2001  2002-2006  2007-2011  2012-2016 | 132  88  201  219  126 | Ref.  0.4 [0.1-1.7]  1.3 [0.6-3.0]  1.4 [0.6-3.1]  1.9 [0.9-4.3] |
| **Females** | 96 |  |
| Period of diagnosis  1991-1996  1997-2001  2002-2006  2007-2011  2012-2016 | 8  9  20  37  22 | Ref.  0.2 [0.0-3.3]  1.5 [0.6-3.9]  1.4 [0.4-4.6]  **3.6 [1.2-10.4]** |

OPC: Oropharyngeal carcinoma; BOT: Base of the tongue; RR: Relative Risk; CI: Credibility interval. ^a^Adjusted by age, gender, period of diagnosis, subsite, tobacco and alcohol consumption. In bold those estimates showing a clear association with HPV-DNA/p16^INK4a^ positivity (i.e. credibility intervals do not contain 1.0).

**Supplementary Table 7. Time trend analysis for HPV positivity (HPV-DNA/p16^INK4a^) in tonsillar cancer patients, with period as continuous.**

|  | **TONSIL samples** | | | |
| --- | --- | --- | --- | --- |
|  | **No. (%)** | **RR^a^** | **[95% CI]** | **Probability^b^ %** |
| **Age at diagnosis**  ≤ 60  > 60 | 182 (52.0)  168 (48.0) | **1.8**  Ref. | [**1.2-2.7**] | 99.7 |
| **Gender**  Male  Female | 302 (86.5)  47 (13.5) | Ref.  1.4 | [0.8-2.2] | 90.0 |
| **Period of diagnosis**  Five-years | 350 (100.0) | **1.3** | [**1.1-1.6**] | 99.8 |
| **Tobacco use**  Non Smoker  < 20 cigarettes/day  ≥ 20 cigarettes/day | 46 (13.7)  54 (16.1)  235 (70.1) | **4.5**  **2.4**  Ref. | [**2.6-7.8**]  [**1.3-4.4**] | 100.0  99.8 |
| **Alcohol consumption**  Non Drinker  <100 grams/day  ≥100 grams/day | 91 (27.0)  95 (28.2)  151 (44.8) | **5.4**  **6.8**  Ref. | [**2.0-14.7**]  [**2.5-18.4**] | 99.95  99.99 |
| **Stage (7th edition)**  I&II  III  IVa  IVb  IVc | 89 (25.7)  72 (20.7)  152 (43.8)  27 (7.8)  7 (2.0) | Ref.  **2.5**  2.0  1.4  0.6 | [**1.2-5.1**]  [0.9-4.0]  [0.5-4.0]  [0.0-19.9] | 99.2  97.1  75.3  38.6 |
| **AIC** | 210.4 | | | |

RR: Relative Risk; CI: Credibility interval. ^a^Adjusted by age, gender, period of diagnosis, subsite, tobacco and alcohol consumption. ^b^Probability of an increasing relative risk. In bold those estimates showing a clear association with HPV-DNA/p16^INK4a^ positivity (i.e. credibility intervals do not contain 1.0).

**Supplementary Table 8. Time trends analysis for HPV positivity (HPV-DNA/p16^INK4a^) in base of the tongue cancer patients, with period as continuous.**

|  | **BOT samples** | | | |
| --- | --- | --- | --- | --- |
|  | **No. (%)** | **RR^a^** | **[95% CI]** | **Probability^b^ %** |
| **Age at diagnosis**  ≤ 60  > 60 | 100 (53.2)  88 (46.8) | 2.4  Ref. | [0.9-6.8] | 95.1 |
| **Gender**  Male  Female | 168 (88.9)  21 (11.1) | Ref.  **2.5** | [**1.1-5.7**] | 98.5 |
| **Period of diagnosis**  Five-years | 189 (100.0) | **1.7** | [**1.1-2.4**] | 99.6 |
| **Tobacco use**  Non Smoker  < 20 cigarettes/day  ≥ 20 cigarettes/day | 21 (12.1)  18 (10.3)  135 (77.6) | **3.7**  **3.7**  Ref. | [**1.4-9.9**]  [**1.4-9.9**] | 99.4  99.4 |
| **Alcohol consumption**  Non Drinker  < 100 grams/day  ≥ 100 grams/day | 31 (17.6)  58 (33.0)  87 (49.4) | 5.7  2.9  Ref. | [1.3-25.5]  [0.6-14.1] | 98.9  91.2 |
| **Stage (7th edition)**  I&II  III  IVa  IVb  IVc | 17 (9.0)  38 (20.1)  100 (52.9)  28 (14.8)  6 (3.2) | Ref.  2.9  3.1  0.4  0.5 | [0.3-26.5]  [0.3-27.3]  [0.0-11.0]  [0.0-15.3] | 83.3  84.2  29.5  34.3 |
| **AIC** | 72.6 | | | |

BOT: Base of the tongue; RR: Relative Risk; CI: Credibility interval. ^a^Adjusted by age, gender, period of diagnosis, subsite, tobacco and alcohol consumption. ^b^Probability of an increasing relative risk. In bold those estimates showing a clear association with HPV-DNA/p16^INK4a^ positivity (i.e. credibility intervals do not contain 1.0).

**Supplementary Table 9. Time trend analysis for HPV positivity (HPV-DNA/p16^INK4a^) in OPC male patients, with period as continuous.**

|  | **Male OPC samples** | | | |
| --- | --- | --- | --- | --- |
|  | **No. (%)** | **RR^a^** | **[95% CI]** | **Probability^b^ %** |
| **Age**  ≤ 60  > 60 | 399 (52.2)  366 (47.8) | **2.1**  Ref. | [**1.3-3.2**] | 99.9 |
| **Period**  Five-years | 766 (100.0) | **1.3** | [**1.1-1.5**] | 99.5 |
| **Tobacco use**  Non Smoker  < 20 cigarettes/day  ≥ 20 cigarettes/day | 58 (8.2)  102 (14.4)  548 (77.4) | **4.8**  **2.6**  Ref. | [**2.7-8.4**]  [**1.5-4.6**] | 100  99.9 |
| **Alcohol**  Non Drinker  < 100 grams/day  ≥ 100 grams/day | 114 (16.0)  229 (32.2)  368 (51.8) | **4.0**  **4.9**  Ref. | [**1.7-9.7**]  [**2.1-11.3**] | 99.9  99.99 |
| **Subsite**  Tonsil  BOT  Tonsil&BOT  Others^b^ | 302 (39.4)  168 (21.9)  17 (2.2)  279 (36.4) | **3.3**  1.8  0.4  Ref. | [**1.6-6.9**]  [0.8-4.2]  [0.0-7.7] | 99.9  91.9  25.9 |
| **Stage (7th edition)**  I&II  III  IVa  IVb  IVc | 167 (21.9)  164 (21.5)  341 (44.7)  75 (9.8)  16 (2.1) | Ref.  **4.5**  **3.0**  1.9  0.7 | [1.7-11.7]  [1.2-7.6]  [0.5-6.5]  [0.0-24.8] | 99.9  99.0  83.4  41.0 |
| **AIC** | 274.0 | | | |

OPC: Oropharyngeal carcinoma; BOT: Base of the tongue; RR: Relative Risk; CI: Credibility interval. ^a^Adjusted by age, gender, period of diagnosis, subsite, tobacco and alcohol consumption. ^b^Probability of an increasing relative risk. In bold those estimates showing a clear association with HPV-DNA/p16^INK4a^ positivity (i.e. credibility intervals do not contain 1.0).

**Supplementary Table 10. Time trend analysis for HPV positivity (HPV-DNA/p16^INK4a^) in OPC female patients, with period as continuous.**

|  | **Female OPC samples** | | | |
| --- | --- | --- | --- | --- |
|  | **No. (%)** | **RR^a^** | **[95% CI]** | **Probability^b^%** |
| **Age at diagnosis**  ≤ 60  > 60 | 56 (59.0)  39 (41.1) | 1.4 | [0.8-2.4] | 86.8 |
| **Period of diagnosis**  Five-years | 96 (100.0) | **1.7** | [**1.2-2.4**] | 99.95 |
| **Tobacco use**  Non Smoker  < 20 cigarettes/day  ≥ 20 cigarettes/day | 32 (34.0)  13 (13.8)  49 (52.1) | **3.5**  2.0  Ref. | [**1.5-7.9**]  [0.7-5.6] | 99.9  91.5 |
| **Alcohol consumption**  Non Drinker  < 100 grams/day  ≥ 100 grams/day | 47 (50.0)  15 (16.0)  32 (34.0) | **14.9**  3.6  Ref. | [**1.9-118.1**]  [0.3-47.1] | 99.5  83.6 |
| **Subsite**  Tonsil  BOT  Tonsil&BOT  Others^b^ | 47 (49.0)  21 (21.9)  2 (2.1)  26 (27.1) | 2.1  **3.4**  0.9  Ref. | [0.7-6.1]  [**1.2-9.7**]  [0.0-64.6] | 91.3  98.9  47.4 |
| **Stage (7th edition)**  I&II  III  IVa  IVb  IVc | 16 (16.7)  20 (20.8)  51 (53.1)  6 (6.3)  3 (3.1) | Ref.  0.9  1.2  1.0  0.2 | [0.5-1.6]  [0.6-2.5]  [0.6-1.5]  [0.0-3.7] | 37.8  73.2  42.7  14.1 |
| **AIC** | 80.9 | | | |

OPC: Oropharyngeal carcinoma; BOT: Base of the tongue; RR: Relative Risk; CI: Credibility interval. ^a^Adjusted by age, gender, period of diagnosis, subsite, tobacco and alcohol consumption. ^b^Probability of an increasing relative risk. In bold those estimates showing a clear association with HPV-DNA/p16^INK4a^ positivity (i.e. credibility intervals do not contain 1.0).

**Supplementary Figure 1. Flow chart of samples disposition and testing for HPV-related biomarkers**


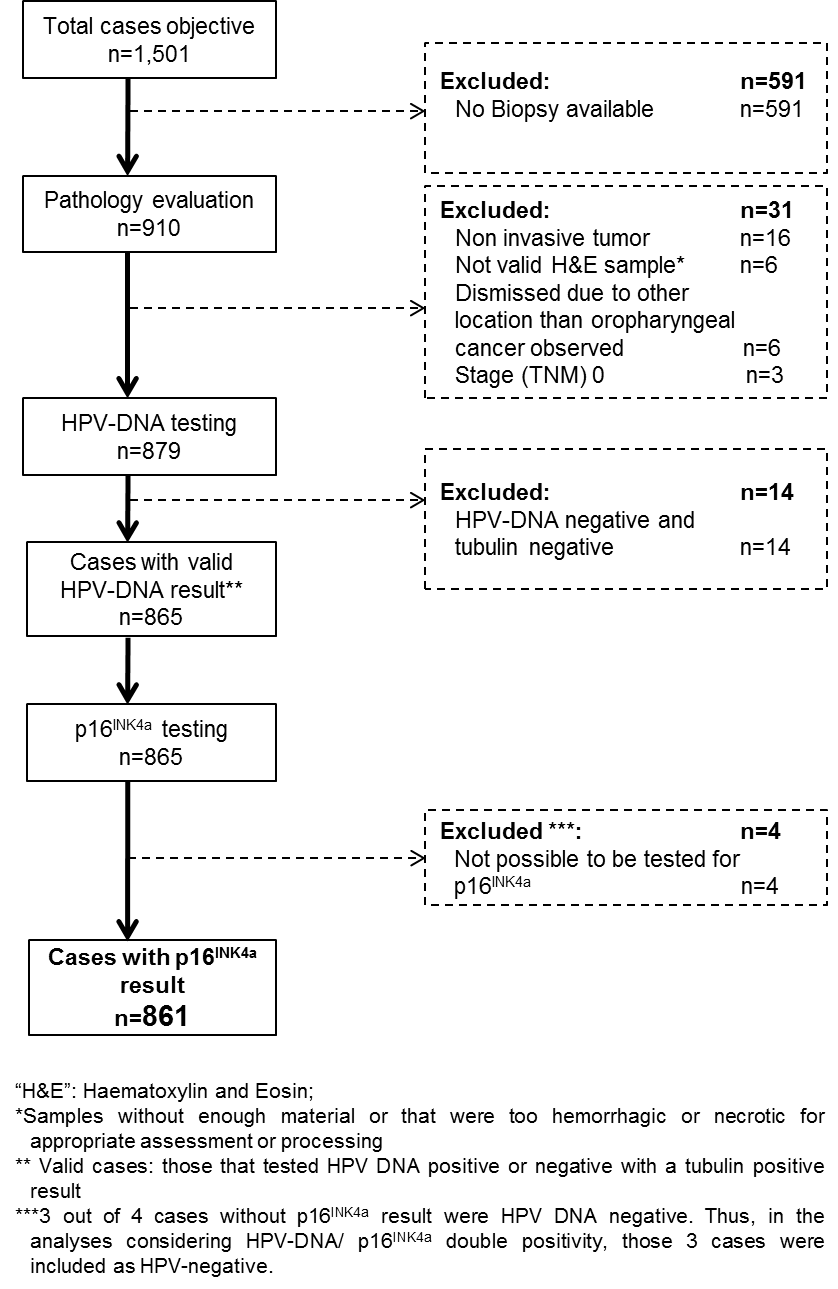


**Supplementary Figure 2. Changes in HPV and smoking prevalence across calendar periods in US and Spain**

**
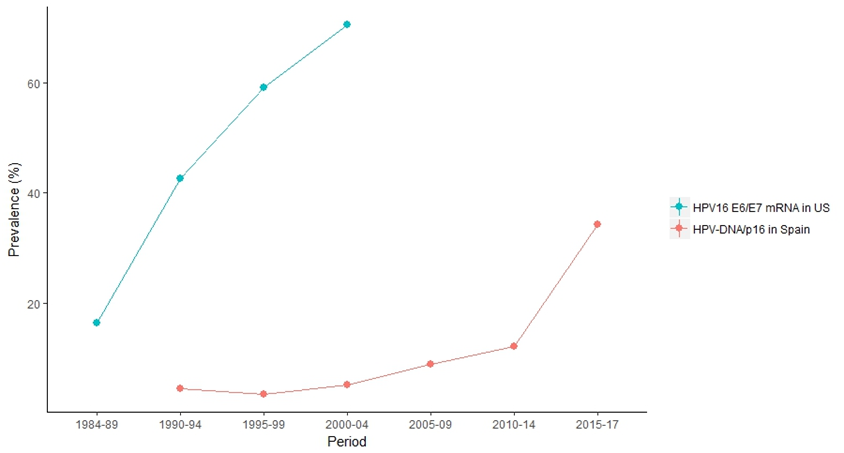
** **
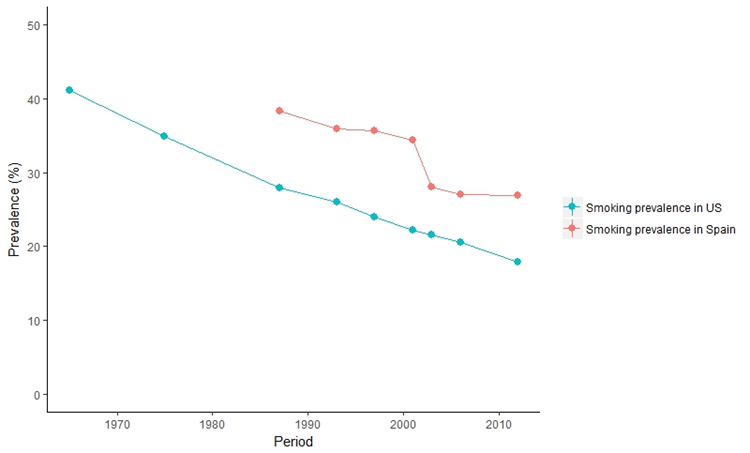
**

Data from US have been adapted from: Chaturvedi et al, J Clin Oncol 2011 [ref 3] for HPV prevalence; and from Centre for Disease Control and prevention:

<https://www.cdc.gov/tobacco/data_statistics/tables/trends/cig_smoking/index.htm> (Accessed Oct 10 2018) for smoking prevalence.
